# Cardiovascular disease risk calculators to reflect the subclinical atherosclerosis of coronary artery in rheumatoid arthritis: a pilot study

**DOI:** 10.1186/s41927-021-00213-3

**Published:** 2021-08-30

**Authors:** Se Hee Kim, Sang-Heon Lee, Hae-Rim Kim, Hong Ki Min

**Affiliations:** 1 Division of Rheumatology, Department of Internal Medicine, Konkuk University Medical Center, 120-1 Neungdong-ro (Hwayang-dong), Gwangjin-gu, Seoul, 05030 Republic of Korea; 2 Division of Rheumatology, Department of Internal Medicine, Research Institute of Medical Science, Konkuk University Medical Center, Konkuk University School of Medicine, Seoul, 05030 Republic of Korea

**Keywords:** Rheumatoid arthritis, Cardiovascular diseases, Atherosclerosis, Coronary artery calcium score

## Abstract

**Background:**

Cardiovascular diseases (CVDs) are the leading cause of death in patients with rheumatoid arthritis (RA). Coronary artery calcium (CAC) score quantifies the severity of atherosclerosis. We estimated CVD risk using several methods and compared these with the CAC score to identify the most suitable CVD risk calculator in RA patients.

**Methods:**

We recruited RA patients, and the 10-year CVD risk was assessed using various tools, viz. Framingham risk score, Systemic Coronary Risk Evaluation (SCORE), Atherosclerotic Cardiovascular Disease (ASCVD) risk estimator plus, QRISK3, Expanded Risk Score in Rheumatoid Arthritis (ERS-RA), and Reynolds risk score. Computed tomography was used to determine the CAC score of each patient. Correlation analysis and linear regression analysis between the CAC score and CVD risk score was performed.

**Results:**

In total, 54 RA patients were enrolled. ERS-RA showed the highest correlation coefficient (*r* = 0.430, *P* = 0.001). In multivariate linear regression analysis, ERS-RA (β = 10.01, 95% confidence interval 3.78–16.23) showed a positive association with the CAC score in RA patients.

**Conclusions:**

The ERS-RA method was highly correlated with the CAC score in RA patients. Therefore, the application of the ERS-RA method may be suitable for predicting subclinical atherosclerosis and CVD risk in RA patients.

**Supplementary Information:**

The online version contains supplementary material available at 10.1186/s41927-021-00213-3.

## Background

Rheumatoid arthritis (RA) is an autoimmune-mediated inflammatory arthritis affecting 0.5–1% of the population [1]. Small joint synovitis is the most frequent symptom in patients with RA, and several synthetic and biologic disease-modifying antirheumatic drugs are used to control the inflammatory response in RA to prevent structural damage of the joints [2]. Pathologic immune cells, such as helper T cells, cytotoxic T cells, autoantibody-producing plasma cells, and macrophages, are known to be involved in the pathogenesis of RA [2]. These pathologic cells cause synovitis and pannus formation in joints, and also induce an inflammatory response in extra-articular organs or tissues, such as the lungs, skin, eyes, or coronary arteries. RA is a systemic autoimmune-mediated disease, and thus, RA patients can have both articular and extra-articular symptoms, which can significantly reduce the quality of life and increase medical expense.

The leading cause of mortality in RA patients is cardiovascular disease (CVD) [3]. The hazard ratio of CVD was found to be 1.94 in RA patients when compared to that in the general population [4]. The European League against Rheumatism (EULAR) taskforce published guidelines for CVD risk management in RA patients [5]. The first step involves estimating the individual risk of CVD with an accurate CVD risk calculator [5]. The estimated CVD risk is then multiplied by 1.5 in RA patients [5]. Several RA-specific CVD risk calculators, such as Expanded Cardiovascular Risk Prediction Score for RA (ERS-RA) and QRISK have been validated in RA patients [6, 7]. However, RA-specific CVD risk calculators were not found to be superior to other traditional CVD risk calculators used in the general population in predicting CVD in RA patients [8].

Coronary artery calcium (CAC) score is a useful method to detect subclinical atherosclerosis in an asymptomatic population. Only low dose radiation (0.7–3 mSv) without contrast enhancement cardiac computed tomography (CT) is required to calculate the CAC score. The CAC score quantifies the severity of atherosclerosis by calculating the area and the density of calcium deposit in the major coronary arteries [9]. This score could provide additional information in patients with an intermediate risk of CVD, which would aid in deciding preventive therapy in these patients [10]. Therefore, the recent American and European Cardiology Associations recommend considering evaluation of the CAC score to improve CVD risk assessment [11, 12]. A study showed that the Framingham risk score significantly correlated with the CAC score in RA patients [13]. However, the most suitable CVD risk calculator for predicting subclinical atherosclerosis in RA patients has not been determined yet. The present study aimed to find out most suitable CVD risk calculator on prediction of subclinical atherosclerosis of RA patients for the first time.

In the present study, we assessed the CVD risk score using different widely used calculators (Framingham risk score, Systemic Coronary Risk Evaluation [SCORE], Atherosclerotic Cardiovascular Disease [ASCVD] risk estimator plus, QRISK3, ERS-RA, Reynolds risk score) in RA patients, and compared these among different RA subgroups based on the CAC score. To identify the most suitable CVD risk calculator in predicting the CAC score, a correlation analysis between the CVD risk score and the CAC score was performed.

## Methods

### Study population

Data of patients who visited single tertiary hospital (Konkuk University Medical Center) between March 2020 and December 2020 were collected. The inclusion criteria were as follows: 1) age between 40 to 79 years, 2) those who fulfilled the American College of Rheumatology-EULAR classification criteria for RA, 3) the CAC score was estimated by computed tomography (CT) for routine medical examination. Patients with a previous history of CVD, other autoimmune diseases, malignancies, or current infection were excluded. The following demographic data were collected from medical charts: gender, age, height, weight, blood pressure, disease duration, comorbidities (hypertension, diabetes mellitus [DM], dyslipidaemia, chronic kidney disease, psychological disorder, migraine, and erectile dysfunction), family history of premature coronary heart disease, and smoking history. In addition, laboratory findings including, rheumatoid factor (RF), anti-cyclic citrullinated peptide (CCP) antibody, total cholesterol, high-density lipoprotein cholesterol (HDL-C), low-density lipoprotein cholesterol (LDL-C), triglyceride, erythrocyte sedimentation rate (ESR), high sensitivity C-reactive protein (hsCRP), and RA-related parameters (Disease Activity Score [DAS] 28-ESR, DAS28-CRP, Clinical Disease Activity Index [CDAI], and modified Health Assessment Questionnaire [mHAQ]) were collected at the same time when the CAC score was estimated by CT. The present study was conducted in accordance with the Declaration of Helsinki and Good Clinical Practice guidelines and was approved by the Institutional Review Board (IRB) of Konkuk University Medical Center (IRB no: 2020–12-002). The requirement for informed consent was waived by the IRB owing to the retrospective design of the study.

### Estimation of the CAC score by CT

**Fig. 1:**
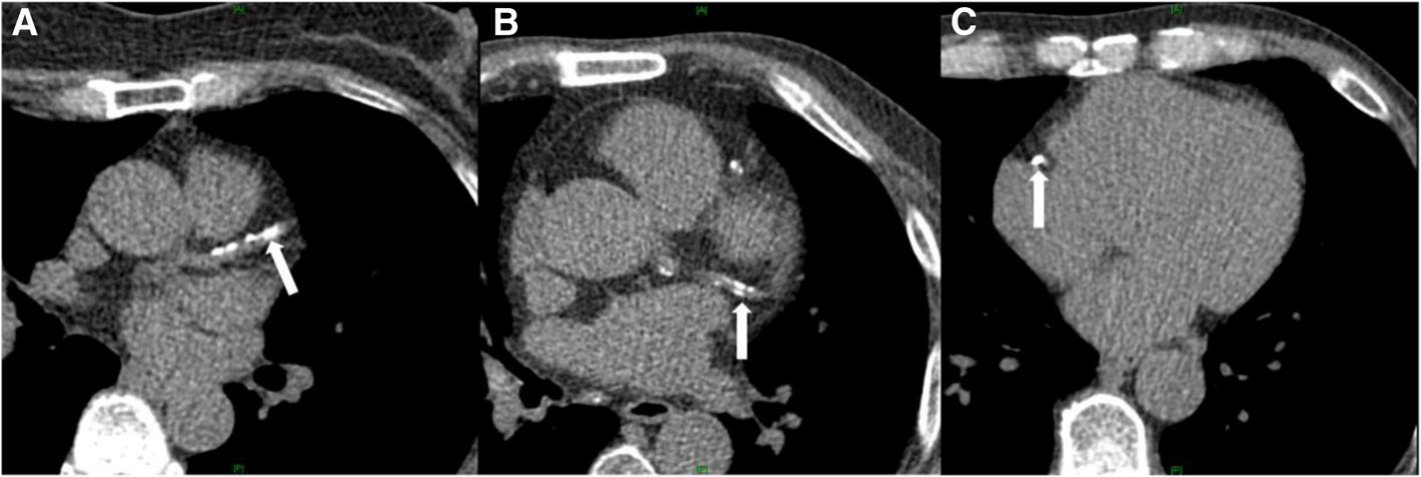
Computed tomography (CT) of coronary arteries. A representative calcium deposit image of coronary arteries detected in a CT scan of the heart. The white arrows indicate the calcium deposit in (**a**) left anterior descending, (**b**) left circumflex, and (**c**) right coronary artery

CT scans were performed with a SOMATOM Force (Siemens, Germany) using the standardized protocol: 3-mm thick, automatic kV selection under ECG-triggering, and breath-holding. The CAC score was quantified using the Agatston method by multiplying the density score and foci in the main coronary arteries [9]. A representative image of calcium deposits in the main coronary arteries are presented in Fig. 1.

### Cardiovascular disease risk score estimation

For the assessment of CVD risk, we selected seven scoring systems, viz. Framingham risk score [14], SCORE for the low and high-risk regions in Europe [15], ASCVD risk estimator plus [11], QRISK3 [7], ERS-RA [6], and Reynolds Risk Score [16]. The CVD risk calculator used in the general population (Framingham risk score, SCORE, ASCVD risk estimator plus, Reynolds risk score) were multiplied by 1.5 according to the EULAR recommendation [5]. Detailed information and included variables of each CVD risk assessment tools are presented in supplementary Table 1.

### Statistical analyses

Continuous variables were presented as the mean ± standard deviation or median with interquartile range. Kolmogorov-Smirnov test was performed testing the normality of the distribution. Spearman analysis was used for evaluating the correlation between the CAC score and the CVD risk score. Univariate and multivariate linear regression analysis was performed to find the factors affecting the CAC score. Factors with *P* value under 0.05 in univariate regression analysis were included in multivariate regression analysis. In all analyses, a *P*-value of < 0.05 was considered statistically significant. All analyses were performed using the software SPSS statistical package (version 25.0 for Windows, SPSS, Chicago, IL, USA).

## Results

### The baseline characteristics of enrolled RA patients

In total, 54 RA patients were enrolled in the final analyses, and all patients were Korean. Mean age of enrolled patients with RA was 60.4 year old, and 24.1% were male. All information of medication, laboratory data, cardiovascular risk estimations were summarized in Table 1.

**Table 1:** Baseline characteristics of enrolled RA patients

Variable	Total RA patients (*N* = 54)
Age	60.4 ± 10.5
Gender (male, %)	13 (24.1%)
BMI	23.0 [21.5; 26.1]
Systolic blood pressure (mmHg)	128.8 ± 14.9
Diastolic blood pressure (mmHg)	74.7 ± 10.0
Disease duration (years)	7.3 ± 7.6
Hypertension	15 (27.8%)
Diabetes Mellitus	5 (9.3%)
Dyslipidemia	18 (33.3%)
Smoking status	
Never smoker	40 (74.1%)
Ex-smoker	4 (7.4%)
Current smoker	10 (18.5%)
Premature angina or heart attack in a 1st degree relative	2 (3.7%)
RF positivity	44 (81.5%)
Anti-CCP positivity	42/53 (79.2%)
ESR (mm/h)	18.5 [4.0; 32.0]
hsCRP (mg/dL)	0.1 [0.0; 0.3]
Total cholesterol (mg/dL)	188.3 ± 41.2
HDL-C (mg/dL)	64.3 ± 19.0
LDL-C (mg/dL)	100.1 ± 36.1
Triglyceride (mg/dL)	106.0 [69.0; 154.0]
Atherogenic index of plasma	−0.1 ± 0.3
Biologics	16 (29.6%)
TNF-α inhibitor	4 (7.4%)
JAK inhibitor	6 (11.1%)
Tocilizumab	5 (9.3%)
Abatacept	1 (1.9%)
Methotrexate	40 (74.1%)
Sulfasalazine	18 (33.3%)
Hydroxychloroquine	21 (38.9%)
Tacrolimus	5 (9.3%)
Leflunomide	5 (9.3%)
Oral glucocorticoid	38 (70.4%)
DAS28-ESR	2.4 [1.4; 3.2]
DAS28-CRP	1.4 [1.1; 2.2]
CDAI	3.0 [2.0; 6.0]
mHAQ	0.0 [0.0; 0.1]
CAC score	0.0 [0.0; 16.7]
Framingham risk score	3.2 [1.2; 9.0]
SCORE for low risk region	1.5 [0.0; 3.0]
SCORE for high risk region	3.0 [1.5; 4.5]
ASCVD risk estimator plus	8.2 [3.4; 19.4]
QRISK3	13.4 [5.9; 22.1]
ERS-RA	7.7 [3.8; 14.4]
Reynolds Risk Score	2.0 [1.0; 4.0]

Continuous variables were presented with mean ± standard deviation or median with interquartile range

*BMI* Body mass index, *DAS28* Disease activity score-28, *CDAI* Clinical Disease Activity Index, *mHAQ* Modified Health Assessment Questionnaire, *CAC* Coronary artery calcium, *SCORE* Systemic Coronary Risk Evaluation, *ASCVD* Atherosclerotic Cardiovascular Disease, *ERS-RA* Expanded Cardiovascular Risk Prediction Score for Rheumatoid Arthritis

### Correlation between CVD risk estimation and the CAC score in RA patients

**Fig. 2:**
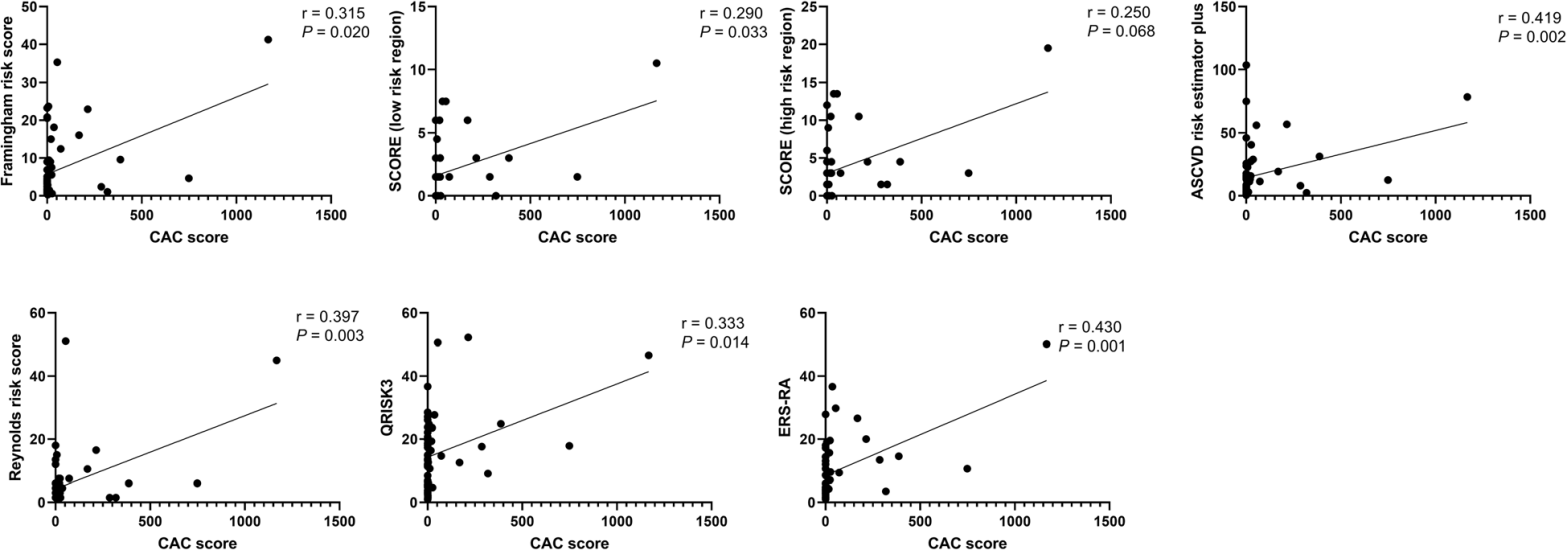
Correlation between 10-year cardiovascular disease risk score and coronary artery calcium score in rheumatoid arthritis patients. Spearman correlation coefficient was calculated between coronary artery calcium (CAC) score and Framingham risk score/Systemic Coronary Risk Evaluation (SCORE)-low-risk region/SCORE-high risk region/Atherosclerotic Cardiovascular Disease (ASCVD) risk estimator plus/QRISK3/Expanded Cardiovascular Risk Prediction Score for rheumatoid arthritis (ERS-RA)/Reynold risk score

To estimate the best-matched CVD risk score with the CAC score, we estimated the Spearman correlation coefficient between each CVD risk score and the CAC score. Framingham risk score, SCORE for the low-risk region, ASCVD risk estimation plus, QRISK3, ERS-RA, and Reynolds risk score showed a significant correlation with the CAC score (Fig. 2). The highest correlation coefficient was found with ERS-RA (*r* = 0.430, *P* = 0.001). Therefore, we selected ERS-RA as one of the variables to be included in the subsequent linear regression analysis for CAC score prediction.

### Predictors of CAC score in RA patients

In the univariate linear regression analysis for predicting the CAC score in RA patients, age (β = 5.67, 95% confidence interval [CI] 0.63–10.71), DM (β = 247.17, 95% CI 70.53–423.80), ERS-RA (β = 10.87, 95% CI 6.02–15.72), and DAS28-ESR (β = 37.55, 95% CI 1.16–73.94) showed positive associations with the CAC score. Variables with a *P*-value of < 0.05 in the univariate analysis were included in the multivariate regression analysis. In the multivariate regression analysis, only the ERS-RA score showed a significant positive association with the CAC score (β = 10.01, 95% CI 3.78–16.23, Table 2).

**Table 2:** Univariate and multivariate linear regression analysis for predicting coronary artery calcium score in RA patients

	Univariate			Multivariate		
	β	95% CI	***P***	β	95% CI	***P***
Age	5.67	0.63, 10.71	0.028	−1.31	−7.09, 4.48	0.652
Male gender	68.53	−58.56, 195.63	0.284			
BMI	−2.89	−15.94, 10.16	0.658			
Disease duration (years)	1.40	− 5.88, 8.68	0.701			
Hypertension	80.00	−40.64, 200.63	0.189			
Diabetes mellitus	247.17	70.53, 423.80	0.007	152.61	−10.80, 316.03	0.067
Dyslipidemia	−69.67	−184.60, 45.26	0.229			
Current smoker	91.26	−47.89, 230.41	0.194			
Premature angina or heart attack in a 1st degree relative	−69.28	− 359.58, 221.01	0.634			
Oral glucocorticoid	91.34	−26.27, 208.95	0.125			
ERS-RA	10.87	6.02, 15.72	< 0.001	10.01	3.78, 16.23	0.002
DAS28-ESR	37.55	1.16, 73.94	0.043	25.11	−6.24, 56.45	0.114

*BMI* Body mass index, *ERS-RA* Expanded Cardiovascular Risk Prediction Score for Rheumatoid Arthritis, *CI* Confidence interval, *DAS28* Disease activity score-28

* All variables yielding *P* value under 0.05 in univariate regression analysis were included in multivariate analysis

## Discussion

The treatment goal of RA is as follows: 1) control synovitis to attain disease remission, 2) prevent joint destruction, 3) control extra-articular symptoms, and 4) prevent atherosclerotic CVD [17]. The CVD risk is increased in RA patients as compared to that in the general population and it is the most common cause of mortality in RA patients [3, 17]. Therefore, proper assessment of CVD risk is crucial to determine the strategy for primary prevention of CVD in RA patients. Atherosclerosis is enhanced by an inflammatory response and oxidative stress [18, 19], and RA patients have a higher inflammatory burden and oxidative stress than the general population. Therefore, EULAR recommends multiplying the CVD risk score by 1.5 if the original CVD risk calculator does not include RA as one of the independent risk factors [5]. ERS-RA and QRISK3 are RA-specific CV risk estimation methods. ERS-RA includes disease duration of RA, RA disease activity, and glucocorticoid use as individual variables for CV risk calculation [6]. On the other hand, QRISK3 includes several newly known CV risk factors, such as RA, erectile dysfunction, migraine, atypical antipsychotic drug use, and glucocorticoid use [7]. These CVD risk prediction methods were developed because patients with RA have higher a CVD risk than the general population and about 30% of CVDs were found to be attributed to RA-specific characteristics [20]. However, a study showed that RA-specific CVD risk calculators were not superior to the traditional CVD risk predictors in predicting CVD in RA patients [8]. Hence, debates still exist on which CVD risk predictor is most appropriate to use in RA patients.

The CAC score was suggested as a CT-based method to evaluate atherosclerotic burden [9]. Previous studies demonstrated that the CAC score correlated with autopsy or intracoronary ultrasound-proven atherosclerotic plaque of coronary arteries [21, 22], implying the excellence of the CAC score in estimating the atherosclerosis of coronary arteries. Furthermore, CT-based CAC score has the advantage of quantifying the calcium deposit in coronary arteries and visualizing the severity of atherosclerosis, wherein the data can be visualised by both physician and patient. The CAC score showed a predictive value for CVD occurrence and was found to be even superior to the Framingham risk score [23, 24]. Furthermore, the CAC score could modify CVD risks, which was originally obtained by the Framingham risk score alone, and the modified CVD risk by CAC score more accurately predicted the actual CVD occurrence than the Framingham risk score, especially in patients with an intermediate risk [25].

Aspirin and statins are generally used for the primary prevention of CVD. A multi-ethnic study on atherosclerosis investigated the role of the CAC score in discriminating patients on statin treatment and showed that statin treatment could be decided based on the CAC score [26]. In addition, the CAC score could reclassify about half of the patients from being eligible to ineligible for statin therapy, especially those with an intermediate risk of CVD (ASCVD risk estimator plus score 5–20%) [27]. In terms of prophylactic aspirin therapy, patients with a CAC score of 0 showed harm with aspirin use, whereas those with a CAC score of ≥100 showed net benefits with aspirin, regardless of other risk factors [28]. Therefore, the clinical importance of the CAC score in the prediction and primary prevention of CVD is growing; the American and European guidelines for CVD prevention recommend the use of the CAC score [10]. To the best of our knowledge, for the first time, this is the first study to demonstrate that ERS-RA best correlated with subclinical atherosclerosis indicated by the CAC score in RA patients.

The present study has several limitations. First, the sample size of the enrolled RA patients was relatively small, and all patients were Koreans. The CVD risk prediction and the CAC score differed depending on the ethnicity of the patients. Further studies involving a larger sample size and participants from multiple ethnicities could clarify the results of this present study. Second, the study was pilot and cross-sectional in nature. Therefore, we presented the usefulness of ERS-RA in predicting subclinical atherosclerosis; however, the utility of ERS-RA in predicting actual CVD occurrence could not be determined from the present study. Third, several factors which could impact on ability of CV risk management such as levels of education, and daily physical activity of patients were not recorded. Finally, the inclusion of a healthy control group could have allowed the selection of a better CVD risk estimation method.

## Conclusions

In conclusion, we demonstrated that ERS-RA highly correlated with the CAC score in RA patients. This finding could strengthen the clinical usefulness of ERS-RA in predicting CVD risk in RA patients and suggest ERS-RA as the most suitable CVD risk predictor in these patients.

## Supplementary Information

**Additional file 1.** Supplemental material for this article is available online.

## Data Availability

The datasets used and analysed during the current study are available from the corresponding author upon reasonable request.

## Abbreviations

RA: Rheumatoid arthritis
CVD: Cardiovascular disease
EULAR: European League Against Rheumatism
ERS-RA: Expanded Cardiovascular Risk Prediction Score for RA
CAC: Coronary artery calcium
CT: Computed tomography
DM: Diabetes mellitus
RF: Rheumatoid factor
anti-CCP: Anti-cyclic citrulinated peptide
HDL-C: High density lipoprotein cholesterol
LDL-C: Low density lipoprotein cholesterol
ESR: Erythrocyte sedimentation rate
hsCRP: High sensitivity C-reactive protein
DAS28: Disease Activity Score 28
CDAI: Clinical Disease Activity Index
mHAQ: Modified Health Assessment Questionnaire
IRB: Institutional Review Board
SCORE: Systemic Coronary Risk Evaluation
ASCVD: Atherosclerotic Cardiovascular Disease
