# Supplementary material for: Cardiovascular disease risk calculators to reflect the subclinical atherosclerosis of coronary artery in rheumatoid arthritis: a pilot study
Source: BMC Rheumatol. 2021 Aug 30;5:39. doi: 10.1186/s41927-021-00213-3 (PMC8404264; doi:10.1186/s41927-021-00213-3)
Supplement: Supplementary file 1 — Additional file 1. Supplemental material for this article is available online. [file 41927_2021_213_MOESM1_ESM.docx]

Supplementary Table 1. Risk factors included in each cardiovascular disease risk calculators

| Variables included | Framingham risk score | SCORE for low and high risk region | ASCVD risk estimator plus | QRISK3 | ERS-RA | Reynolds Risk Score |
| --- | --- | --- | --- | --- | --- | --- |
| Age | ● | ● | ● | ● | ● | ● |
| Sex | ● | ● | ● | ● | ● | ● |
| Race |  |  | ● | ● |  |  |
| Systolic Blood Pressure | ● | ● | ● | ● |  | ● |
| Diastolic Blood Pressure |  |  | ● |  |  |  |
| Height |  |  |  | ● |  |  |
| Weight |  |  |  | ● |  |  |
| Total Cholesterol | ● | ● | ● | ● |  | ● |
| HDL-C | ● |  | ● | ● |  | ● |
| LDL-C |  |  | ● |  |  |  |
| CRP |  |  |  |  |  | ● |
| Hypertension or treated with medication | ● |  | ● | ● | ● |  |
| Diabetes Mellitus |  |  | ● | ● | ● |  |
| Dyslipidemia or Statin user |  |  | ● |  | ● |  |
| Smoking | ● | ● | ● | ● | ● | ● |
| Aspirin |  |  | ● |  |  |  |
| Premature angina or heart attack in a 1^st^ degree relative |  |  |  | ● |  | ● |
| Chronic Kidney Disease |  |  |  | ● |  |  |
| Atrial fibrillation |  |  |  | ● |  |  |
| RA |  |  |  | ● |  |  |
| RA duration > 10 yrs |  |  |  |  | ● |  |
| CDAI |  |  |  |  | ● |  |
| mHAQ |  |  |  |  | ● |  |
| Systemic lupus erythematosus |  |  |  | ● |  |  |
| Severe mental illness |  |  |  | ● |  |  |
| Migraine |  |  |  | ● |  |  |
| Glucocorticoid |  |  |  | ● | ● |  |
| Atypical antipsychotic drug |  |  |  | ● |  |  |
| Erectile dysfunction |  |  |  | ● |  |  |

SCORE, Systemic Coronary Risk Evaluation; ASCVD, Atherosclerotic Cardiovascular Disease; ERS-RA, Expanded Cardiovascular Risk Prediction Score for Rheumatoid Arthritis; HDL-C, high density lipoprotein cholesterol; LDL-C, low density lipoprotein cholesterol; CRP, C-reactive protein; RA, rheumatoid arthritis; CDAI, Clinical Disease Activity Index; mHAQ, modified Health Assessment Questionnaire
